# A Rare Case of Melorheostosis in the Hand of a Saudi Woman

**DOI:** 10.7759/cureus.8877

**Published:** 2020-06-28

**Authors:** Abdulaziz H Abed, Hosam T Mashrah, Akeel M Almahdaly, Mahmood Shaheen

**Affiliations:** 1 Medicine and Surgery, Alfaisal University College of Medicine, Riyadh, SAU; 2 Medicine and Surgery, Taif University, Taif, SAU; 3 Orthopaedics, King Saud Medical City, Riyadh, SAU; 4 Orthopaedics, King Faisal Specialist Hospital and Research Centre, Riyadh, SAU

**Keywords:** rare, case, melorheostosis, leri’s disease

## Abstract

Melorheostosis is a very rare bone dysplasia, especially in the hand. Most cases were diagnosed incidentally, with the lower limbs being the most affected. This is the first Saudi woman with hand melorheostosis.

A 33-year-old Saudi female had mild to moderate right-hand pain that started six years ago. Hand examination showed a full range of motion and full hand grip, and there was no tenderness upon palpation. Plain X-ray, unenhanced CT scan, and MRI of the hand showed an appearance resembling dripping candle wax as melorheostosis. The bone scan showed a nonvascular and nonacute lesion. An unenhanced CT scan demonstrated cortical and endosteal hyperostosis involving the proximal, middle, and distal third and fourth phalanges. Multi-sequential MRI of the hand demonstrated cortical hyperostosis involving the ulnar and radial aspect of the right fourth proximal, middle, and distal phalanges.

Features in the X-ray, CT scan, bone scan, and MRI confirmed a diagnosis of melorheostosis with associated flexor tenosynovitis.

## Introduction

Melorheostosis is a very rare bone dysplasia, especially in the hand with limited reports in the literature. It has no clear etiology with an equal incidence rate among males and females, and its main features involve dermal, soft tissue abnormalities, and the anomalous bone formation [1-2]. The incidence of melorheostosis is 0.9 cases per million population, and in 50% of cases, were diagnosed before the age of 20 [3]. It is a benign process without associated mortality but leads to functional limitations. Its etiology and etiopathogenesis are unknown [4].

Patients commonly present with pain, deformities, limitations of a range of motion, contractures, muscle atrophy, and limb swelling [5]. Most cases were diagnosed incidentally and may involve different skeletal regions such as vertebrae, sternum, or upper and lower extremities, with the lower limbs being the most affected [1, 6-7]. Complications such as trigger finger or carpal tunnel syndrome, along with joint stiffness causing motion range limitation and deformities, could develop due to soft tissue swelling and fibrosis. In some cases, it causes joint deformities that may become so severe as to require surgical treatment [1, 8].

Melorheostosis is diagnosed through standard radiographs, which show a linear area of osteosclerosis along with the affected bones, producing a dripping candle wax appearance [5]. This case study presents a case of melorheostosis diagnosed and treated at King Faisal Specialist Hospital and Research Centre. The clinical aspect and management of this condition are discussed with a review of the relevant literature.

## Case presentation

A 33-year-old Saudi female patient who was medically free and in her usual state of health until about six years ago started to have mild to moderate right-hand pain. According to her, the pain comes and goes with 4/10 in severity, no aggravating or releveling factors. Pain is not affecting her life. History was negative for weight loss, fever, or night sweats. 

**Figure 1:**
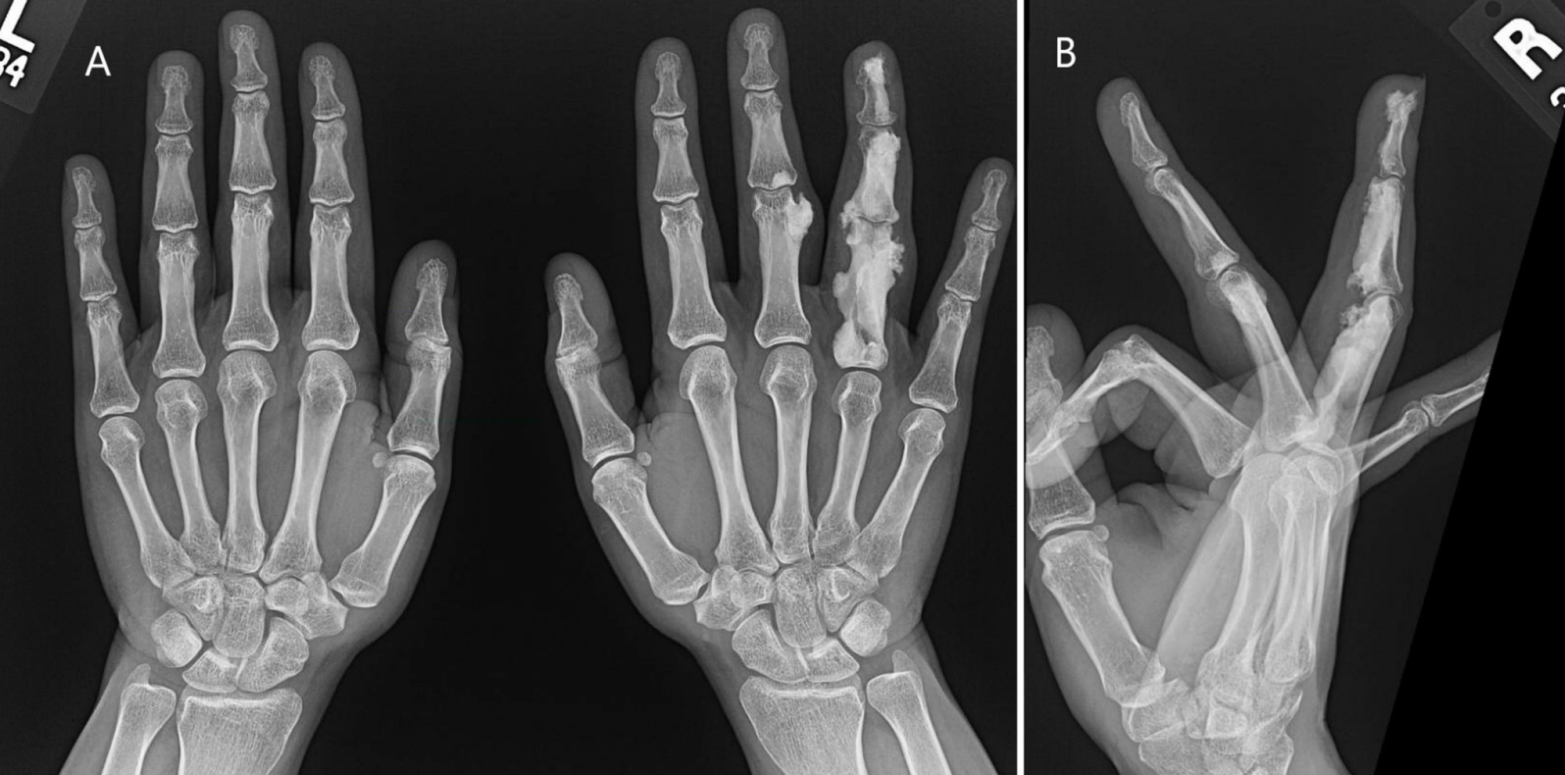
Plain X-ray: A - Bilateral hands. B - Right hand.

Hand examination showed a full range of motion and full hand grip, and there was no tenderness upon palpation. Laboratory tests were regular. A plain X-ray of the right hand was obtained in posteroanterior and oblique views, as well as a dedicated lateral view for the ring finger. Wavy hyperostosis was noted involving the ulnar as well as the radial aspect of the fourth proximal, middle, and distal phalanges as well as the ulnar aspect of the third proximal and middle phalanges with extension to the soft tissue that showed soft tissue swelling. The appearance resembles dripping candle wax appearance, most likely representing melorheostosis (Figure 1).

**Figure 2:**
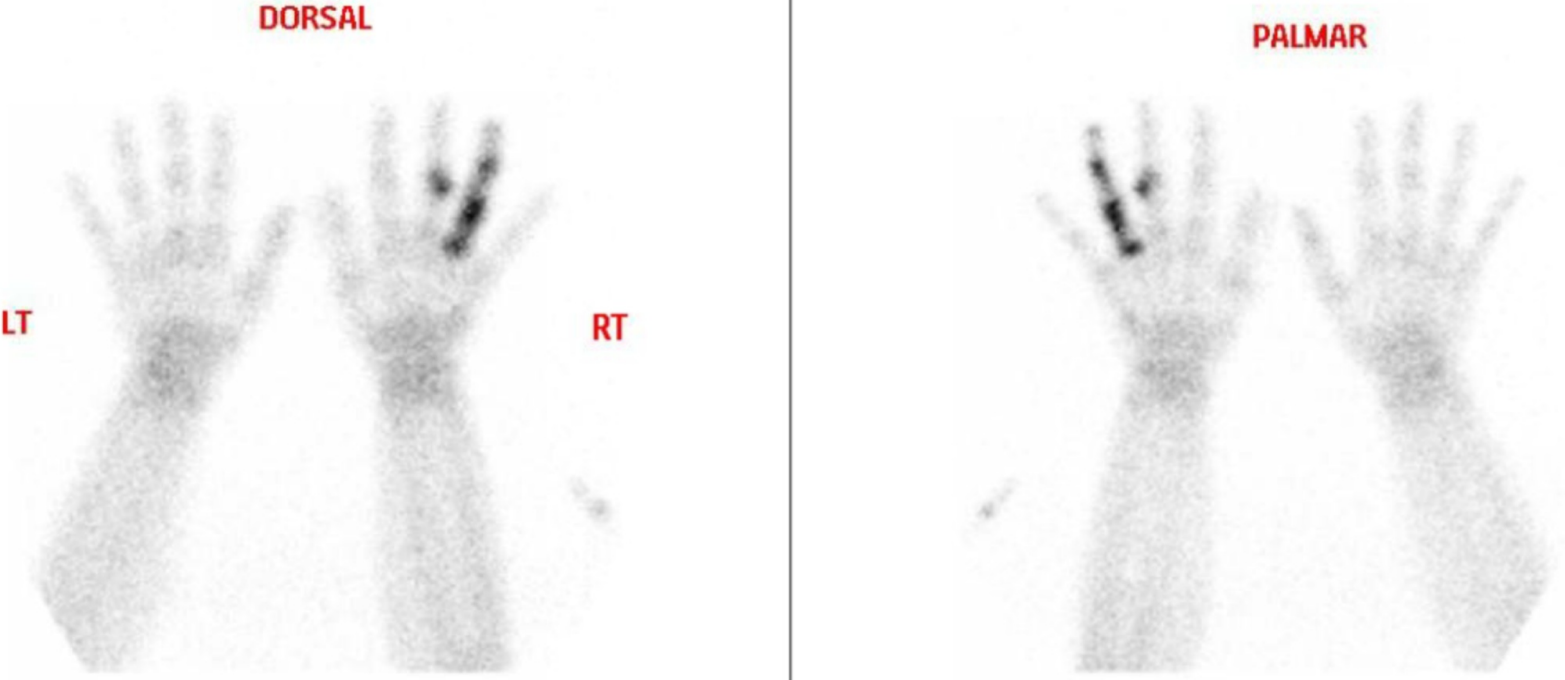
Bone scan of the hands.

A bone scan of the hands followed total body bone scan. The left fourth proximal, medial, and distal phalanx and the left third middle phalanx showed intense tracer uptake in the bone phase with no corresponding increased tracer uptake in the flow in blood pool images indicating nonvascular lesions. The rest of the whole body skeleton shows no abnormal, localized tracer uptake. The described lesions were nonvascular and nonacute (Figure 2).

**Figure 3:**
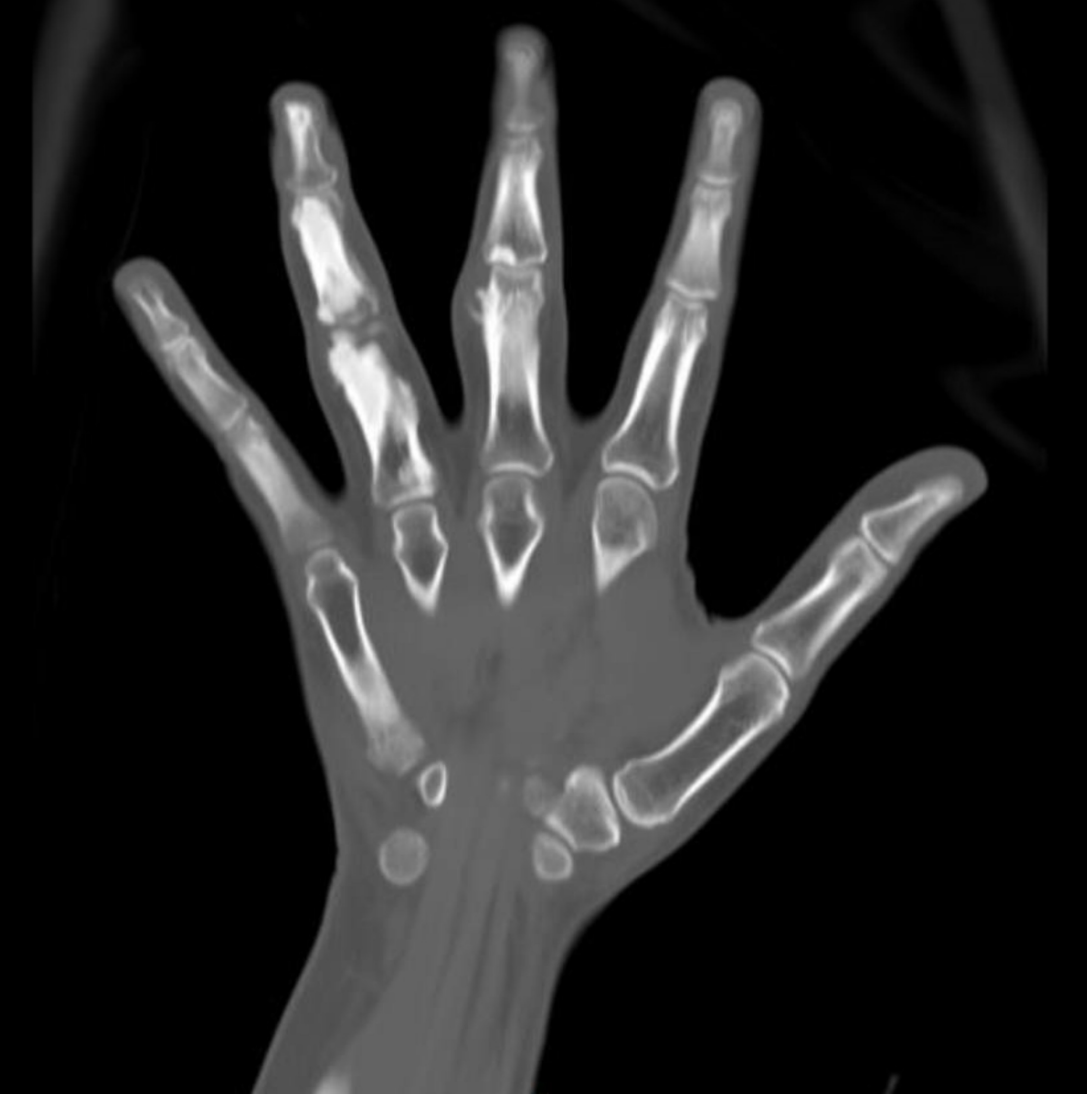
CT scan of the right hand.

An unenhanced CT scan of the right hand was obtained in control, axial, and sagittal reformats and demonstrated cortical and endosteal hyperostosis involving the proximal, middle, and distal third and fourth phalanges. It showed mild adjacent soft tissue bulge. However, the adjacent interphalangeal joints appeared unremarkable with no visible deformity. The above features were suggestive of the melorheostosis (Figure 3).

**Figure 4:**
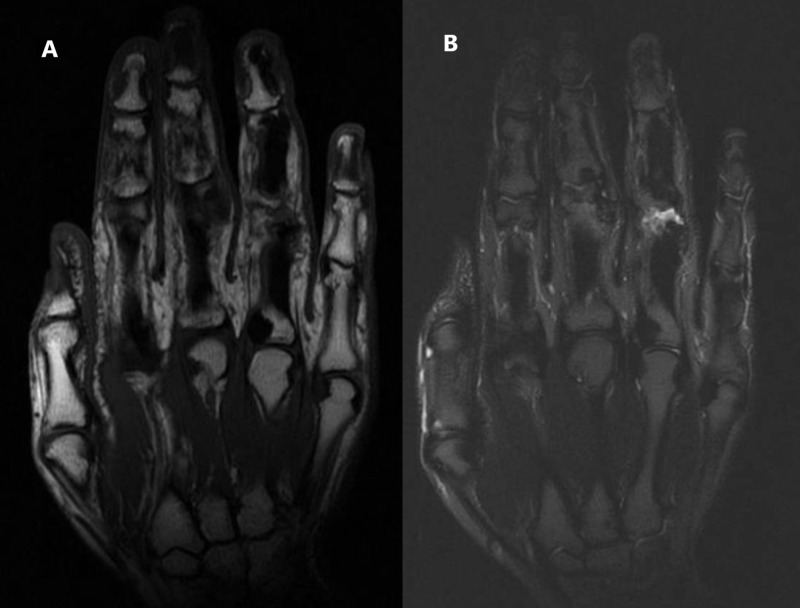
MRI of the right hand. A) T1-Weighted MRI. B) T2-Weighted MRI.

Multi-sequential MRI of the hand was obtained with tumor protocol and demonstrated cortical hyperostosis involving the ulnar and radial aspect of the right fourth proximal, middle, and distal phalanges. There appears low signal in all sequences, and there was a minimal extraosseous extension, especially at the volar aspect with subsequent mild soft-tissue edema and swelling. Less pronounced changes involved the third proximal phalanx and middle phalanx with minimal soft tissue extension and bulge at the ulnar aspect. There was associated minimal joint effusion and synovial thickening at the third and fourth proximal interphalangeal joint as well as features of the flexor tenosynovitis of the ring finger (Figure 4).

With a previous X-ray and CT scan, the above features were reported, and a diagnosis of melorheostosis with associated flexor tenosynovitis was given. No active treatment was needed for the patient. She was referred to physiotherapy, which reduced her pain. During her four years follow up at our hospital, there were no changes in her symptoms or the size of her hand, also no change in the radiological images.

## Discussion

Leri’s disease or melorheostosis was first described in 1922. It is a rare mesodermal bony dysplasia that may involve surrounding soft tissue [9]. Its classic appearance in the plain radiograph is a dripping wax of periosteal cortical thickening [10-11]. Pain, deformity, limitation in range of motion, and limb swelling are the most common presentation. It equally affects both sexes and diagnosed incidentally, as reported in numerous studies in adults and children.

The gene mutation in MAP2K1 was discovered in patients who were diagnosed with melorheostosis [12]. Lab investigations were found to be insignificant during diagnosis [12]. Until 2012 the etiology was not known, but the X-ray finding was given and well known. Also, the treatment of that patient was symptomatic without any surgical interventions [13]. 

A study has shown that the gene mutation was given in MAPK1, causing “Wax appearance” and suggested that MEK1/2 inhibitors are the future treatment in such conditions [5]. A previously rare case of melorheostosis was reported, where the patient was free of chronic conditions or diseases and had mild to moderate right-hand pain that was not affecting his daily life activities. Local examination of the hand showed a full range of motion and a full hand grip without any limitation. Also, the presentation was reported to be free medically with normal examination and range of motion [14].

Diagnosis of melorheostosis may be confirmed by radiological studies such as X-rays, CT, MRI, and bone scan [15]. Bone scintigraphy, MRI, or CT is helpful and was found to delineate the severity of the illness and help the physician to operate better [16].

In the case presented in this study, the patient showed disclosed wavy hyperostosis in both ulnar and radial aspects of the fourth proximal, middle, and distal phalanges. Also, only the ulnar aspect of the third proximal and middle phalanges with extension to the soft tissue and swelling as well. The appearance resembles dripping candle wax, which made the diagnosis for us after excluding other differential diagnoses. The differential diagnosis in such a case should be considered, which are infection like myositis ossificans or malignancy like periosteal osteosarcoma or osteopoikilosis [17]. And for unclear presentation, the biopsy could be recommended [14].

We present a case report of a rare pathology in unusual site affection with no previous reports in studies or literature in Saudi Arabia. Regardless of the incidence globally, it is recommended for researchers, readers, and physicians to be familiar with good knowledge about the uniqueness of the disease. It is always better to diagnose it early to avoid comorbidity and operative complications. The lack of knowledge and awareness of similar cases of rare diseases could lead to misdiagnosis and unexpected complications.

The presented case needed no active treatment; however, in other cases, melorheostosis is a benign dysplasia that can trigger severe morbidity. Melorheostosis morbidity usually tends to be due to pain, contractures, limitation of joint motion, differences in length of limb, and bone deformities. Treatment of melorheostosis includes medical treatment for symptom control and soft surgical treatment complication in tissues [18].

## Conclusions

This is a rare case of a 33-year-old Saudi female with mild to moderate right-hand pain that started six years ago. Dripping candle wax appearance was found in plain X-ray, bone scan showed nonvascular lesion, CT scan showed cortical and endosteal hyperostosis of the phalanges, and MRI showed cortical hyperostosis of the ulnar and radial aspect of the right fourth proximal, middle, and distal phalanges. All reported features confirmed a melorheostosis diagnosis.
